# EHMTI-0121. The disturbances of reactivity of cerebral vessels in patients with posttraumatic headaches

**DOI:** 10.1186/1129-2377-15-S1-C23

**Published:** 2014-09-18

**Authors:** I Iakubenko, T Litovchenko, O Zavalna

**Affiliations:** 1 Neurology, Kharkov Medical Academy of Postgraduate Education, Kharkov, Ukraine

## 

Microstructural disturbances develop in the wall of vessels in patients with mild traumatic brain injuries (TBI).

The purpose of our research was to detect the regularities of changes of hemodynamics and reactivity of cerebral circulation in patients with consequences of mild traumatic brain injuries by using the method of Doppler ultrasonography with hyper-and hypocapnic tests.

## Materials and methods

We examined 62 patients with consequences of mild TBI. The control group consisted of 20 healthy people. Doppler ultrasonography with hyper-and hypocapnic tests were carried out according to the standard method.

## Results

Conducting Doppler ultrasonography with hyper-and hypocapnic tests in the patients with consequences of TBI we detected that coefficient of reactivity was 1.11±0.07. The insignificant asymmetry of bloodflow which increased after hyper- and hypocapnia was observed in the patients (82%). An early development of atherosclerotic lesion of cerebral vessels in the form of hemodynamic insignificant atherosclerotic plaques of the walls of cerebral vessels was observed (73%). We detected paradoxical reaction of cerebral vessels in 40 patients when hyper-and hypocapnic tests were being held.

## Conclusions

The data obtained confirm the changes of reactivity of cerebral vessels towards the decrease of linear velocity of bloodflow in the patients with consequences of mild TBA. The detected paradoxical reaction of vessels which was observed when we used hyper-and hypocapnic tests, is the consequence of the decrease of elasticity of vessels walls as a result of microstructural disturbances of vessels, an early development of atherosclerotic changes in vessels and the manifestation of a long-term angiospasm.

No conflict of interest.

